# Impact of health education intervention on knowledge and perception of cervical cancer and cervical screening uptake among adult women in rural communities in Nigeria

**DOI:** 10.1186/1471-2458-14-814

**Published:** 2014-08-07

**Authors:** Olumide A Abiodun, Oluwatosin O Olu-Abiodun, John O Sotunsa, Francis A Oluwole

**Affiliations:** Department of Community Medicine, Benjamin Carson (Snr) College of Medicine, Babcock University, Ilishan, Nigeria; The School of Nursing, Ijebu-Ode, Ogun State Nigeria; Department of Obstetrics and Gynaecology, Benjamin Carson (Snr) College of Medicine, Babcock University, Ilishan, Nigeria; Department of Community Medicine and Primary Care, Obafemi Awolowo College of Health Sciences, Olabisi Onabanjo University, Sagamu, Nigeria

**Keywords:** Cervical cancer, Cervical screening, Knowledge, Perception, Awareness, Participatory health education, Movie

## Abstract

**Background:**

Cervical cancer is a disease of public health importance affecting many women and contributing to avoidably high levels of cancer deaths in Nigeria. In spite of the relative ease of prevention, the incidence is on the increase. This study aimed to determine the effect of health education on the awareness, knowledge and perception of cervical cancer and screening among women in rural Nigerian communities.

**Methods:**

The study design was quasi-experimental. The study was carried out among adult women in Odogbolu (intervention) and Ikenne (control) local government areas (LGA) of Ogun state. Three hundred and fifty (350) women were selected per group by multistage random sampling technique. Data was collected by semi structured interviews with the aid of questionnaire. The intervention consisted of structured health education based on a movie.

**Result:**

The intervention raised the level of awareness of cervical cancer and screening to 100% (p < 0.0001). The proportion of women with very good knowledge of cervical cancer and screening rose from 2% to 70.5% (*χ*^2^ = 503.7, p < 0.0001) while the proportion of those with good perception rose from 5.1% to 95.1% (p < 0.0001). The mean knowledge and mean perception scores were also increased (p < 0.0001). There was increase in the proportion of women who had undertaken cervical screening from 4.3% to 8.3% (p = 0.038). The major reason stated by the women for not having had cervical screening done was lack of awareness about cervical cancer and screening. There was statistically significant difference between the intervention and control groups concerning their knowledge attitude and practice towards cervical and screening (p < 0.05) after the intervention.

**Conclusion:**

Multiple media health education based on a movie is effective in creating awareness for and improving the knowledge and perception of adult women about cervical cancer and screening. It also improves the uptake of cervical cancer screening. The creation of awareness is very crucial to the success of a cervical cancer prevention programme.

## Background

Cervical cancer is the malignant neoplasm of the cervix uteri. Globally, there are nearly 1.5 million cases of clinically recognized cervical cancer [1]. Eighty five percent (85%) of these are in developing countries like Nigeria. While industrialized countries have reduced its incidence by over 70% in the last 50 years, the burden seems to be on the rise in less developed countries [1]. It is expected that the incidence of cervical cancer in developing countries will rise from 444,546 to 588,922 between 2012 and 2025 [1].

The most important risk factor is Human Papillomavirus (HPV) infection, whereas lack of accessible cervical screening services is a major barrier to screening uptake. Other risk factors are early age at sexual contact, early marriage (below age 20 years), multiple partners, polygamy, multi-parity and lack of awareness of the disease [2]. Cervical cancer is attended by huge financial and social burden. It is a social disease especially of the poor and less educated in whom the risk factors are most prevalent. Nigeria is extrapolated to lose between 347.4 and 482.7 million US Dollars each year to cancers [3]. Cancer of the cervix can be prevented by providing widespread and regular cervical screening services for all women who have been sexually active. This is done by the HPV test, Pap test or the Visual Inspection of the Acetic Acid painted cervix (VIA) which is affordable and more sensitive [4]. Vaccination of women against the HPV before the onset of sexual activity also prevents the disease [4]. However, this is very expensive at the moment. The One-Visit Approach – screening with VIA by trained personnel and provision of cryotherapy for obvious mild to moderate cervical dysplasia is recommended for developing countries [4].

In Nigeria, 40.43 million women are at risk of developing cervical cancer [5]. Current estimates indicate that every year, 14,089 women are diagnosed with cervical cancer and 8,240 die from the disease [1]. About 23.7% of women are estimated to harbour cervical HPV infection while over 90% of invasive cervical cancers are attributed to HPV subtypes 16 or 18 [5]. It is projected that in 2025, there will be 19,440 new cervical cancer cases and 10,991 cervical cancer deaths in Nigeria [1]. Cervical cancer was found to be the commonest cancer of women in many parts of Nigeria [6, 7] and has a national age standardized incidence rate of 33.0 cases per 100,000 women per year [1]. However, the level of awareness is quite low. Only about 15% of women aged 20-65 yrs in the south-west region of Nigeria have heard about the disease [8]. Majority (60%) of the population live in rural areas with no access to cervical screening. Currently in Nigeria, less than 10% of women have ever had cervical screening [5], whereas, 40 to 50% of women are screened in developed countries [8].

Nigeria does not have a well-articulated and widely disseminated National Cervical Cancer Policy and there is no widespread cervical screening for women [9]. Therefore, women come to hospitals with invasive cancer of the cervix at advanced stages when radiotherapy is of little or no benefit and even radical hysterectomy is of no benefit [7]. There is a pressing need for accessible and affordable screening services. This is particularly important because at least 70.8% of Nigerians live on less than US$1 per day [5]. It is an investment into the nations’ and families’ fortunes and future [10].

The World Health Organization supported a study of the effectiveness and acceptability of VIA and Cryotherapy in six African countries including Nigeria [9]. In the Nigerian project site, 100 healthcare workers in 49 health facilities in Ogun State were trained and equipped to undertake VIA in their community settings. During the period between September 2007 and May 2010, a total of 5,529 women were screened for cervical cancer. Large variations were observed in the implementation of screening programmes in the various facilities across Local Government Areas (LGA). Indeed, at more than half of the health facilities, fewer than two women were screened per month on the average. Over this period, only 118 women were screened in Odogbolu local government area and one of the three VIA centres in the local government area did not have any screening done at all [9].

Videos as a medium of health education have proved to be invaluable visual aids with high levels of effectiveness when used as health education tools in many different settings [11–14]. In recent times, the home video industry has thrived in Nigeria and the populace appears to have a greater preference for local films depicting their culture and tradition. Women and Children have been observed to spend much time watching these videos either in their homes or in the neighborhood. They are attracted more to these videos because they are cultural, colorful, and watched in a relaxed atmosphere [15]. A culturally appropriate health education video in the Yoruba language titled “Asunle*”* was developed and targeted at women, young girls and indeed the general population. The purpose of the video was to promote the uptake of cervical screening among adult women and promote vaccination among eligible girls by highlighting the risk factors and symptoms of cervical cancer and educating them about ways of preventing cervical cancer.

This study evaluated the effectiveness a home video centered Health Education intervention among Yoruba speaking adult women in a rural LGA in Nigeria.

## Methods

### Study design and study area

The study design is Quasi-experimental- before and after study. The Study was carried out in Odogbolu (intervention) and Ikenne (control) Local Government Areas (LGA) of Ogun State. Ikenne (control) LGA is similar to the intervention LGA in all respects. Odogbolu LGA is divided into fifteen (15) wards. At an annual growth rate at 2.8% for the country, the projected Population as at 2013 is about 154,233. There are twenty three health centres, three of which are equipped for VIA in the intervention LGA. Ikenne LGA has ten wards and a projected population of 144,056 as at 2013. Females constitute 42.1% of the Population [16]. There are eighteen health centres, four of which are equipped for VIA in the control LGA.

### Sampling

Sample Size was determined using the formula for estimating proportions in experimental studies [17]. The aim was to achieve results at 95% confidence interval, confer 80% power and a desired degree of accuracy of 0.05. The estimated proportion was taken as 4.7% which was the proportion of women who had accessed cervical screening services in Ago-Iwoye [18], a town in an LGA adjacent to the intervention LGA. A sample size of 281 was derived per group. An allowance of 20% was made for non response and attrition making a sample size of 337. However, 350 individuals each were recruited in the intervention and control groups.

All consenting women between the ages of 25 and 64 years in Odogbolu LGA were eligible to participate in the study. All consenting women between the ages of 25 and 64 years in Ikenne LGA were eligible to serve as Controls. The ages 25 to 64 years represent the age in which cervical changes are most significant and hence cervical cancer screening most relevant. Cervical changes are normal in women below 25 years [19]. The natural history and progression of cervical cancer show that it is highly unlikely that women who are 65 years and older without a prior positive screening result will go on to develop the disease [19]. Women outside the ages of 25 and 64 years and those who refused to consent were excluded from the study. Three hundred and fifty women between the ages of 25 and 64 years were recruited from each of the intervention and control LGAs. A total of 700 women participated in the study.

A multistage sampling technique was used. Four of the fifteen wards in Odogbolu Local Government Area were randomly selected by balloting. Sample sizes were proportionately allocated to the wards. Systematic random sampling was used to determine houses from which participants were drawn. One household was selected per house using simple random sampling by balloting. One eligible consenting woman was then recruited per household by simple random sampling by balloting. If no eligible woman was found in a household, eligible participants were sought from the next household or house as the case was. The same multistage sampling process outlined above was replicated for Ikenne Local Government Area of Ogun State to select three hundred and fifty (350) women who served as control.

### Data collection instruments

Data was collected with the aid of interviewer administered Questionnaire. The questionnaire assessed the demographic characteristics, awareness of cervical cancer and screening, the risk factors, symptoms and means of prevention of cervical cancer. It also assessed the perception about cervical cancer and screening. The participants were required to correctly identify risk factors, symptoms and means of prevention by responding with ‘yes’, ‘no’ or ‘don’t know’ as appropriate. The questionnaire also investigated the various barriers to cervical cancer screening. The questionnaire was translated to the Yoruba language (the local language) and then back to English to ensure content validity.

### Pre-intervention activities

The research assistants consisted of 4 Health Educators, 10 volunteer final year medical students and 2 senior resident Doctors. Three-day training was organized for the research assistants on the effective communication of the epidemiology, presentation, diagnosis, treatment, prevention and screening of cervical cancer by the researchers. The volunteers were also trained on the study objectives, sampling process and questionnaire administration. The proficiency of the interviewers was verified through pre-testing and the noticed gaps filled. Furthermore, field monitoring was carried out to check quality of data being collected.

The Questionnaire was pre-tested among thirty five (35) women between the ages of 25 and 64 years at Ode-lemo in Sagamu Local Government Area of Ogun State, Nigeria.

A base-line survey to determine the level of awareness, knowledge and perception of cervical cancer screening was conducted using the pre-tested questionnaire. The questionnaire was translated into Yoruba language. The questionnaires were administered by the ten final year medical students. This involved both the intervention and control groups.

### Intervention activities

A structured health education was given to the intervention group respondents. The intervention group received health education on cervical cancer and screening while the control group received education on breast cancer and screening. The control group also received health education on cervical cancer and screening after the post intervention study. The health education received by the control group on breast cancer included didactic lectures, practical sessions and participatory learning sessions.

The investigators used multiple channels to educate the intervention group. Didactic lectures were given. The lecture guides were adapted from the training manual for peer educators designed by the cervical cancer prevention program of Zambia [20] and covered topics on the burden, risk factors, symptoms and prevention of cervical cancer. A twenty five minutes Health Education movie (in Yoruba) on Cancer of the Cervix titled ‘ASUNLE’ was aired to the women. The lectures and movie stimulated the women to ask questions and participatory health education sessions followed. The women were encouraged to answer questions that were raised before the facilitator made clarifications as necessary. The women were then provided with a hand bill (in Yoruba and English languages) on Cervical Cancer and screening to be read at home.

The women were educated in batches of 50. Each batch was trained for one day over a 4 hour period from 10.00 to 14.00 hours. Health education intervention lasted for 7 days.

### Post intervention activities

An immediate post intervention assessment was carried out to confirm that the messages were well understood. The participants were required to answer 20 post-test questions at the end of each training day. Thirteen weeks after the campaign, a post intervention evaluation was conducted using the same interviewer administered questionnaire. The women who had being trained were invited to participate in the post intervention evaluation. The control group was then given the same health education the intervention group had while the intervention group received health education on breast cancer. The women were then encouraged to access the VIA services at the VIA centres within the LGAs.

### Data analysis

The Data obtained from the questionnaire were stored in a computer and analyzed using the Statistical Package for Social Sciences Software (SPSS 15.0). Knowledge and perception scoring were done by assigning one (1) mark for each correct knowledge and perception questions respectively. The maximum knowledge score was 40; scores from 0 to 14 were designated as ‘very poor’, scores from 15 to 19 were designated as ‘poor’, scores from 20 to 24 were designated as ‘good’ while scores greater than 24 were designated as ‘very good’. The maximum perception score was 5; scores from 0 to 2 were designated as ‘poor’ while scores from 3 to 5 were designated as ‘good’. This scale is similar to that used by Ogun and Bejide [21].

The data was summarized using proportions, means and standard deviation. The Fisher’s exact test, chi square and students’t test were used to determine associations as appropriate.

### Ethical consideration

The study was approved by the Olabisi Onabanjo University Teaching Hospital Ethical Committee. Permissions were obtained from the Chairmen of Odogbolu and Ikenne LGAs, and the community leaders. The research participants were enrolled in the study after written informed and voluntary consent. Confidentiality was maintained.

## Results

At post intervention, 325 (92.9%) of intervention and 289 (82.6%) of control group respondents were available to complete the study questionnaire.

Table 1 shows the socio-demographic characteristics of the study participants. There was no statistically significant difference between the intervention and control groups in terms of their socio-demographic characteristics. More than 90% of participants in both intervention and control groups had completed primary school education.

**Table 1:** **Socio-demographic characteristics of participants**

Factors	Experimental group N = 350 (%)	Control group N = 350 (%)	Test statistic value (***χ*** ^2^)	p-value
**Age**				
25-34 years	253 (72.3)	246 (70.3)	0.703	0.873
35-44 years	62 (17.7)	65 (18.6)		
45-54 years	23 (6.6)	28 (8.0)		
55-64 years	12 (3.4)	11 (3.1)		
**Total**	350	350		
**Marital status**				
Married	285 (81.4)	276 (78.9)	4.820	0.185
Single	59 (16.9)	58 (16.6)		
Divorced	2 (0.6)	4 (1.1)		
Widowed	4 (1.1)	12 (3.4)		
**Total**				
**Religion**				
Christianity	251 (71.7)	238 (68.0)	2.263	0.520
Islam	98 (28.0)	109 (31.1)		
Traditional	1 (0.3)	2 (0.6)		
Atheist	0 (0.0)	1 (0.3)		
**Total**	350	350		
**Ethnicity**				
Yoruba	321 (91.7)	324 (92.6)	1.943	0.584
Hausa	8 (2.3)	4 (1.1)		
Igbo	13 (3.7)	16 (4.6)		
Others	8 (2.3)	6 (1.7)		
**Total**	350	350		
**Level of education**				
No formal education	19 (5.4)	24 (6.9)	1.192	0.755
Primary school	110 (31.4)	105 (30.0)		
Secondary school	178 (50.9)	172 (49.1)		
Post-secondary	43 (12.3)	49 (14.0)		
**Total**	350	350		
**Employment status**				
Self employed	267 (76.3)	269 (76.9)	1.142	0.888
Employed	43 (12.3)	40 (11.4)		
Student	11 (3.1)	15 (4.3)		
Unemployed	27 (7.7)	25 (7.1)		
Retired	2 (0.6)	1 (0.3)		
**Total**	350	350		
**Average monthly income (Naira)**				
0- 4,999	95 (27.1)	98 (28.0)	2.129	0.831
5,000- 9,999	115 (32.9)	122 (34.9)		
10,000- 14,999	59 (16.9)	49 (14.0)		
15,000- 19,999	23 (6.6)	21 (6.0)		
20,000- 24,999	29 (8.3)	25 (7.1)		
25,000 and Above	29 (8.3)	35 (10.0)		
**Total**	350	350		

Table 2 shows that among intervention and control groups, the awareness of cervical cancer and screening was low, the knowledge and perception was poor; hence the uptake of cervical screening was quite low at baseline. The vast majority of participants in the intervention (94%) and control groups (93.7%) had very poor knowledge about cervical cancer. The perception was also very poor in both intervention (94.9%) and control (93.1%) groups. However, the vast majority of participants demonstrated willingness to get cervical screening done. There was no statistically significant difference between the two groups at baseline. The median and interquartile range of knowledge scores for both groups was 0.00 each while the median and interquartile range of perception scores for both groups were 1.00 and 0.00 respectively.

**Table 2:** **Participants’ awareness, knowledge and perception of cervical cancer and uptake of cervical screening at baseline**

Variable	Intervention	Control	Test value	p value
Ever heard of cervical cancer	59 (16.9%)	50 (14.3%)		p = 0.404*
Ever heard of cervical screening	36 (10.3%)	38 (10.9%)		p = 0.902*
Ever had cervical screening	15 (4.3%)	12 (3.4%)		p = 0.695*
Willingness to have cervical screening	314 (89.7%)	320 (91.4%)		P = 0.518*
Mean knowledge score	1.75 (SD = 5.65)^α^	2.03 (SD = 5.77)^α^	t = 0.649 (df = 698)	P = 0.517
Mean perception score	1.13 (SD = 0.77)^α^	1.16 (SD = 0.83)^α^	t = 0.496 (df = 698)	P = 0.620

*Fisher’s exact test was used, ^α^mean (SD = standard deviation).

Table 3 shows that post intervention, among intervention, the awareness of cervical cancer and screening was high, the knowledge and perception was relatively better. However, the control group remained poor. The difference between the intervention and control group was statistically significant (p < 0.05). There was no statistically significant difference in the willingness to have cervical screening between the two groups (p = 0.6402).

**Table 3:** **Participants’ awareness, knowledge and perception of cervical cancer and uptake of cervical screening at post intervention**

Variable	Intervention	Control	Test value	p value
Ever heard of cervical cancer	325 (100.0%)	45 (15.6%)		p < 0.0001*
Ever heard of cervical screening	325 (100.0%)	31 (10.7%)		p < 0.0001*
Ever had cervical screening	27 (8.3%)	11 (3.8%)		p = 0.0281*
Willingness to have cervical screening	300 (92.3%)	270 (93.4%)		P = 0.6402*
Mean knowledge score	25.69 (SD = 6.20)^α^	2.22 (SD = 6.04)^α^	t = 47.391 (df = 612)	p < 0.0001
Mean perception score	4.43 (SD = 0.92)^α^	1.17 (SD = 0.88)^α^	t = 44.7316 (df = 612)	p < 0.0001

*Fisher’s exact test was used, ^α^mean (SD = standard deviation).

Table 4 showed that health education had statistically significant effect on the awareness of cervical cancer and screening. The mean knowledge and perception scores were also improved in the intervention group. There was a marked improvement in the proportion of correct answers to specific questions about the cervical cancer risk factors, symptoms, methods of prevention and about cervical screening among the intervention group. However, there was no statistically significant difference among the control group. The perception about cervical cancer was significantly improved among the intervention group, while there was no statistical difference among the control group.

**Table 4:** **The effect of health education among study participants**

Knowledge score (0–40)	INTERVENTION				CONTROL			
	Baseline n = 350 frequency (%)	Post intervention n = 325 frequency (%)	% change	Statistic (P)	Beginning of study n = 350 frequency (%)	End of study n = 289 frequency (%)	% change	Statistic (P)
Ever heard of cervical cancer	59 (16.9)	325 (100.0)	+83.1	(p < 0.0001*)	50 (14.3)	45 (15.6)	+1.3	(p = 0.657*)
Ever heard of cervical screening	36 (10.3)	325 (100.0)	+94.6	(p < 0.0001*)	38 (10.9)	31 (10.7)	−0.2	(p = 1.000*)
Ever had cervical screening	15 (4.3)	27 (8.3)	+4.0	(p = 0.038*)	12 (3.4)	11 (3.8)	+0.4	(p = 0.834*)
Willingness to have cervical screening	314 (89.7)	300 (92.3)	+2.6	(p = 0.283*)	320 (91.4)	270 (93.4)	+2.0	(p = 0.373*)
Mean knowledge score	1.75 ± 5.65^α^	25.69 ± 6.20^α^		t = 52.48 (p < 0.0001)	2.03 ± 5.77^α^	2.22 ± 6.04^α^		t = 0.406 (p = 0.685)
Mean perception score	1.13 ± 0.77^α^	4.43 ± 0.92^α^		t = 50.66 (p < 0.0001)	1.16 ± 0.83^α^	1.17 ± 0.88^α^		t = 0.148 (p = 0.883)

*Fisher’s exact test was used, ^α^mean ± Standard deviation.

The proportion of intervention group participants with very poor knowledge of cervical cancer reduced from 94% to 7.4%, whereas those with very good knowledge increased from 2% to 70.5% (*χ*^2^ = 503.7, p < 0.0001). The proportion of control group participants with very poor knowledge of cervical cancer reduced from 93.7% to 93.1%, whereas those with very good knowledge increased from 2% to 2.4% (*χ*^2^ = 0.159, p = 0.984). This difference was not significant statistically. Likewise, the proportion of intervention group participants with a good perception about cervical cancer rose from 5.1% to 95.1% (p < 0.0001) while it rose from 6.9% to 7.6% among the control group participants (p = 0.760).

Table 4 shows that there was a 4% increase in the uptake of cervical screening among the intervention group (p = 0.038); whereas, the control group remained essentially the same increasing by 0.4%, a change which was not statistically significant (p = 0.834).

The willingness to get cervical screening done was slightly raised in both the intervention (+2.6%) and control groups (+2.0%). However, the increases were not statistically significant (p = 0.283 and p = 0.373 respectively).

Table 5 shows the barriers to the uptake of cervical screening as identified by the study participants. Prior to intervention, lack of awareness of cervical cancer was identified by the majority of participants in both intervention (94%) and control groups (91%) as the main reason why they had not undergone cervical screening. This was followed by lack of knowledge of where to access cervical screening services. However, at post intervention, 96.4% of the participants in the intervention group identified lack of knowledge of where to access screening services as the major barrier (p < 0.0001). There was no statistically significant change among the control group participants (p = 0.6113).

**Table 5:** **Barriers to uptake of cervical screening**

Barriers	INTERVENTION		CONTROL					
	Baseline n = 335 frequency (%)	Post intervention n = 298 frequency (%)	% change	***X*** ^2^(P)	Beginning of study n = 338 frequency (%)	End of study n = 278 frequency (%)	% change	***X*** ^2^(P)
Lack of awareness	315 (94.0)	0 (0.0)	−94		308 (91.1)	247 (88.8)	2.3	
Lack of access to screening services	12 (3.6)	298 (100.0)	96.4		19 (5.6)	17 (6.1)	0.5	
Poor quality of health services	0 (0.0)	0 (0.0)	0	586.7 (P < 0.0001)	2 (0.6)	2 (0.7)	0.1	2.688 (P = 0.6113)
Cost of service	6 (1.8)	0 (0.0)	−1.8		6 (1.8)	5 (1.8)	0	
Lack of interest	2 (0.6)	0 (0.0)	−0.6		3 (0.9)	7 (2.5)	1.6	

## Discussion

The health education intervention used a movie on cervical cancer and screening to stimulate participatory health education. Hand bills produced in both Yoruba (the local language) and English were given to the women to reinforce what had been learnt.

Communication in relation to health education involves different modes like lectures, group or panel discussions, symposia, poster or exhibit presentation [22]. Every individual mode of health education has its own merits, drawbacks as well as its own sphere of effectiveness. In addition it has to overcome the barriers of communication e.g. physiological, psychological, environmental and cultural [22]. A specific mode of communication is more useful in a specific setting and on a specific group than others. The search for the optimum mode of communication for specific audiences is a major area of research in health education. Some of the earlier studies have already stressed the need of exploring the background and character of the recipient group while imparting health education [23]. Some studies have shown the success of different modes of communication in different situations [24–28]. Movie centered health education proved to be very effective in improving awareness, knowledge and perception of cervical cancer and screening among rural women in Nigeria. The uptake of cervical screening was also improved.

The awareness of cervical cancer and screening was remarkably increased among the intervention group at the post intervention stage. All of the respondents reported that had heard about cervical cancer and screening. The control group did not experience significant change in awareness levels for cervical cancer and screening. There was statistically significant difference between the two groups at post intervention. This was not the case at baseline.

There was also significant improvement in the knowledge about cervical cancer and screening in the intervention group. Majority of the respondents were able to associate cervical cancer with a virus. The knowledge of risk factors, symptoms and prevention were also significantly improved. All the respondents in the intervention group remembered that immunization and cervical screening were means of preventing cervical cancer. Many more women (compared to baseline) were able to correctly identify who should have undergone cervical screening and how often cervical screening should be done. Other studies among similar and different groups have shown that appropriately selected health education methods have shown improved awareness and knowledge of cervical cancer and screening and indeed other health issues [29–33]. A study among high school teachers divided in 3 groups: experimental 1 (educated by pamphlets), experimental 2 (educated by a lecture and flash cards), and control group (not manipulated) showed that there were significant differences in mean scores of knowledge and attitude of 2 experimental groups as compared with the control group just like in this study. However, there was also a difference between the 2 experimental groups. Education by lecture and flash cards was more effective than by pamphlets [23]. Another study showed that media led campaigns are also quite successful at improving health awareness among Vietnamese-American women in Alameda and Santa Clara Counties in northern California [34]. Personally delivering education through peer counselors are thought to be a better breast health promotion method than mailing printed educational materials [35].

This study also showed significant improvement in the intervention groups’ perception of cervical cancer. They were better able to appreciate their individual risk of cervical cancer. All the women stated that women with vaginal bleeding should visit the hospital. There was statistically significant difference between the two groups at post intervention. This was not the case at baseline.

This study showed a statistically significant improvement in the uptake of cervical screening among the intervention group, while the control group remained essentially the same. Other studies to assess the impact of health education show conflicting results. Some studies have shown that knowledge concerning HPV, cervical cancer and cervical cancer screening was statistically improved after educational intervention, but the concern about getting cervical cancer was not allayed [33]. Media led campaigns are also quite successful at improving health awareness but may or may not increase the uptake of services [33]. However, other studies have shown the opposite. Screening information conveyed by promoteras (lay health educators) successfully prompted Hispanic women to obtain mammography and Papanicolaou smears [36]. A media-led education intervention succeeded in increasing recognition of and intention to undertake screening tests more than receipt of the tests, though it improved the receipt of the test [34]. Other studies however gave equivocal results. For example a randomized controlled trial compared a photo-comic on cervical cancer screening with a placebo comic. One month after the comics were distributed, a radio-drama paralleling the photo-comic was broadcast on the community radio station and a retrospective evaluation was carried out. The study concluded that the photo-comic was ineffective in increasing cervical screening uptake in this population but that the radio-drama may have had more impact, though only a minority of women recalled being exposed to it [37].

The success of this study at improving uptake of cervical screening may be attributed to the fact that the population had very little prior knowledge of cervical cancer and screening. It is left to be seen whether there will be sustained improvement of cervical screening uptake with time as demonstrated by other interventions that are based on social and behavioural theories [38].

The intervention did not have any statistically significant effect on the willingness to know more about cervical cancer and screening in both intervention and control groups. However, the willingness to know more was high in both groups before and after intervention. The major barrier after intervention was lack of knowledge of where to access screening services as reported by the respondents in the intervention group.

### Limitations of the study

Randomized controlled study is the gold standard for investigating Interventions. The Experiment and control groups were not picked from the same LGA; hence no randomization needed to be done. The study was quasi-experimental in design. The attrition in control group was quite higher than the intervention group probably due to some loss of interest and confusion having received health education on Breast cancer and being asked questions about cervical cancer and screening. However, there were enough post intervention respondents to ensure that the findings of the study were valid.

## Conclusion

Multimedia Health Education based on a movie led to a remarkable improvement in the awareness, knowledge and perception about cervical cancer and screening among adult rural women in Nigeria. Uptake of cervical screening services was also increased. The major barriers to cervical screening were lack of awareness and poor knowledge about cervical cancer and screening. This was followed by lack of knowledge of where to access cervical screening services. Knowledge and perception of cervical cancer and screening in rural communities can be improved by giving appropriate health education intervention. The creation of awareness and improving access to screening services are crucial to the success of a cervical cancer screening programme. The scale up of this intervention with similar result is quite feasible considering the nationwide acceptance of the home video industry in Nigeria.
